# Hospital Emergency Preparedness Plan Review in a Low-Resource Country After a Mass Casualty Incident: The Beirut Blast

**DOI:** 10.7759/cureus.71410

**Published:** 2024-10-14

**Authors:** Ramzi Nakhle, Danielle Abou Khater, Yara A Mouawad, Joelle Kalaji, Mariana Helou

**Affiliations:** 1 Emergency Department, Lebanese American University School of Medicine, Beirut, LBN

**Keywords:** beirut blast, disaster, emergency medicine, emergency preparedness plan, mass casualty incident

## Abstract

A mass casualty incident (MCI) is an event that overwhelms the available medical resources due to a large number of casualties, typically requiring immediate medical attention and exceeding the normal capacity of emergency response systems. MCIs mandate the immediate transition from the daily working routine in a hospital to crisis mode through the implementation of an emergency preparedness plan (EPP). Following an MCI, hospitals must anticipate chaos and a large influx of patients, some with life-threatening injuries that require fast decision-making. Lebanon is a low-resource country that has already been suffering from the consequences of the COVID-19 pandemic and an ongoing economic crisis. This study reviews the EPP of the Lebanese American University Medical Center in response to an MCI that resulted from the “Beirut blast” disaster.

## Editorial

Mass casualty incidents (MCIs), whether natural, such as floods and earthquakes, or man-made, such as terrorist attacks or industrial accidents, come without warning. They mandate an immediate shift from the daily hospital routine to crisis mode through the implementation of an emergency preparedness plan (EPP). In the event of an MCI, hospitals must anticipate a surge of patients, some with life-threatening injuries, requiring rapid decision-making in a chaotic environment.

In recent decades, the Middle East has experienced multiple MCIs due to political and economic instability. Lebanon is a small country in the Middle East with an area of 10,452 km² and an estimated population of about 6.7 million with approximately 1.5 million refugees, mostly from Syria [1-3]. At the time, Lebanon had been suffering from the consequences of the COVID-19 pandemic, increasing poverty rates and unemployment, as well as a deepening economic crisis. Following these events, the healthcare system has crumbled, facing a shortage of medications and medical supplies [4]. On August 4, 2020, the country was further shaken by the massive explosion at the Port of Beirut.

The purpose of this study is to review the EPP of the Lebanese American University Medical Center (LAUMC) in response to an MCI in a low- to middle-income country, following its experience with victims of the Beirut blast. In order to be better prepared to handle future MCIs with more practical strategies, we aim to highlight the aspects of the EPP that were effective and those that were adopted after the devastating event.

Report

The Beirut Blast

On August 4, 2020, at 6:07 PM, two large explosions devastated the Lebanese capital city of Beirut. The first explosion at the Beirut port was followed, 30 seconds later, by a far more destructive blast, resulting in a significant number of casualties and widespread damage throughout the city [4]. Investigations suggest that the blast was triggered by a fire that ignited 2,750 tonnes of highly explosive ammonium nitrate, which had been stored in a warehouse at the port for six years [5]. The explosion led to a massive influx of patients to the emergency departments across the capital: 8,643 were injured, 1,056 were admitted to regular hospital wards, 156 were admitted to the intensive care unit (ICU), 534 underwent surgeries, and 93 died in various hospitals. A month after the blast, reports indicated over 200 fatalities [6].

In addition to the ongoing burden of the COVID-19 pandemic, five university hospitals located near the Beirut port sustained major damage, rendering them nonfunctional and resulting in the loss of over 500 hospital beds that could have been used for patients. Only three university hospitals remained operational to receive and care for patients, particularly those in critical condition [7]. Other non-university hospitals in Beirut were also treating injured individuals. The explosion occurred at 6:07 PM when only a few physicians and nurses were on duty. To address this, a mobile phone instant messaging network was used to send an alert to all doctors, requesting them to report to the hospital by activating the emergency plan, code white [8]. Within less than an hour, total chaos erupted in the emergency department (ED).

The Study Site: Lebanese American University Medical Center

The Lebanese American University Medical Center (LAUMC), established in 1925, is a private teaching university hospital with a capacity of 200 beds and an estimated 15,000 emergency department visits each year. It is located in Beirut, 2.8 km from the Beirut port.

In 2020, while the hospital was undergoing several renovations, the ED was temporarily relocated. The temporary ED had a total of 13 beds, including seven beds inside and five beds outside, placed in isolation chambers created in response to the COVID-19 pandemic [8].

LAUMC Emergency Plan

Based on past experiences with MCIs, a disaster committee (DC) was established, headed by the Chief Medical Officer (CMO). Frequent meetings were held with relevant departments, which conducted a risk assessment study and developed an EPP that was updated periodically. The most recent revision occurred in 2018, with plans to conduct an emergency drill at the hospital [8]. The emergency response strategy addressed all potential risks, focusing primarily on the MCIs that the risk assessment indicated were most likely to occur in the region [9].

First, the EPP emphasized patient identification. Triage staff were instructed to manually enter the patient's full name on a pre-printed numbered label. Recognizing that it would be difficult to register patients in the usual manner during an external disaster, a paper-based registration system was developed for such scenarios. Second, it focused on triage. The Simple Triage and Rapid Treatment (START) [10] tool, with its four color-coded zones (red, yellow, green, and black), was adopted. Patients with life-threatening injuries were to receive immediate care (red zone), followed by those with acute but non-life-threatening injuries (yellow zone), individuals with minor or no physical injuries (green zone), and deceased patients (black zone) [11].

Moreover, the plan addressed communication issues during a disaster. Communication was enabled through Emergency Communication Equipment, including two-way radios that allowed contact between departments such as the ED, the ICU, and the operating room (OR).

The EPP also tackled crowding by monitoring hospital capacity. If the ED surpassed its capacity for receiving more red or yellow triage tag patients, the Command Center would be alerted, and external emergency authorities, such as the Red Cross, would be contacted to transfer patients elsewhere. Additionally, journalists and reporters were to be directed to another facility to gather information on the event, preventing them from accessing the ED.

Response to the Disaster

On the evening of August 4, 2020, a massive explosion occurred in Beirut, inflicting severe damage to the hospital. The blast shattered windows, knocked out lights, and scattered dust throughout the facility. The temporary ED was particularly hard hit, with the roof collapsing, the main ED door jammed shut and shattered glass littering the floor. Although an EPP had been authorized and revised for LAUMC, the hospital faced various challenges, including patient overload, communication issues, and difficulties with patient identification and registration. While the disaster plan helped streamline patient care, its implementation faced unforeseen challenges.

Within the first hour of the incident, over 300 people were brought to the emergency department, most suffering from blast injuries caused by broken glass [7]. All available medical personnel were mobilized to ensure proper triage and treatment. It was late in the evening, and most hospital staff had already left. Staff on duty at the time of the blast included one emergency physician, one resident, and one medical student, along with four registered nurses, two practical nurses, one transporter, and one admissions officer. Two physicians happened to be on the hospital grounds at the time, and two others who lived nearby arrived within minutes. The disaster code was activated by the operator once it became clear that the explosion had originated outside the hospital and the influx of casualties began.

The CMO activated the disaster code and sent a message via WhatsApp Messenger to all available physicians, urging them to come to the hospital to assist. Within 30 minutes, the ED was overwhelmed, with patients lying on beds, on the floor, and even outside. Initially, walking patients with minor lacerations and injuries began to arrive, primarily from the areas surrounding the blast. Soon after, severely injured patients were brought in by car or by the Lebanese Red Cross from closer to the explosion site. This initial period allowed time to assemble the ED medical team to assist with the triage and treatment of the more critically injured. The emergency physician on call was stationed at the back door to initiate triage for the influx of patients and later moved the triage area to the outside of the ED.

Residents and medical students took an expanded role in care and leadership until additional help arrived. The most common injury seen in the ED was lacerations from broken glass, making suturing the most frequently performed intervention that day. Early arrivals were sutured using the appropriate technique, but as the number of patients surged, sutures were replaced by staples, often without anesthetics or adequate sterilization. Fortunately, there were enough supplies to treat every casualty.

Statistics following the explosion at LAUMC revealed that a total of 408 cases came through the ED; 331 of those cases were treated on-site, 72 casualties were admitted to the hospital, nine were admitted to the ICU, and five patients died. A total of 58 cases underwent surgery [8].

Discussion

Emergency Preparedness Plan

Triage: Although the EPP was designed to handle a large influx of patients, the number of casualties far exceeded all predictions, overwhelming any existing plan. Implementing the START triage system proved to be challenging due to the overwhelming number of patients and the severity of their injuries. The sheer volume of patients, coupled with the complexity of the injuries, made it difficult to categorize and prioritize care effectively in a timely manner. This strained the triage process and required significant resources to ensure that critical cases were attended to promptly. Patients were classified based on their condition as either walking/stable, unstable, or deceased. Due to the lack of available space, those who were walking and less severely injured were moved to temporary locations such as the wound care and chemotherapy units for suturing and proper management. Critically injured patients were treated in the ED and transferred to the OR if necessary.

Patient identification: In such a chaotic situation, electronic systems failed, and even pre-printed numbered labels proved neither sufficient nor practical. As a result, most patients who came to the ED were treated and discharged without being documented. Their information was later gathered when they returned for follow-up visits.

Hospital network system: Our EPP included the option of transferring patients to nearby hospitals when necessary. However, on that day, nearby hospitals were damaged, with two of them unable to operate. It was impossible to contact any other facility, and, to make matters worse, roads were blocked, making patient transfers impossible.

Chaos: Communication between staff members was a challenge. Amid the shouting of patients in pain and family members demanding information, instructions for the radiology department were written directly on the patient's chest or arms, indicating the necessary imaging. Similarly, radiologists documented their findings in the same manner.

Infectious disease exposure: In the aftermath of that day, a COVID-19 outbreak was anticipated. Doctors and nurses were treating wounds, intubating patients, and conducting physical exams while wearing only surgical masks, as there was no time to don full personal protective equipment. The medical team had to risk exposure to COVID-19 in order to provide timely care to the injured.

Revision of the EPP

After experiencing the obstacles and flaws in the EPP highlighted by the blast and exacerbated by its occurrence during the COVID-19 pandemic, various adjustments were made to the EPP based on several unstructured discussions with front-line physicians, surgeons, nurses, and other healthcare workers.

Triage: The primary focus is on the necessity of applying mass casualty triage outside of the emergency department to enhance hospital capacity for handling catastrophic injuries. Triage was critical in the disaster response, especially given the enormous number of injuries, inadequate space, and limited personnel. A quick and efficient triage mechanism was needed to address the crisis promptly and effectively. Due to the circumstances, we were unable to carry out the START triage as outlined in the earlier plan, so we modified our triage technique to accommodate the disaster situation. The initial triage level should be conducted outside the ED to differentiate between stable patients and those who require critical care. Any medical staff on the emergency team at the time of the disaster, including nurses, practical nurses, medical students, and physicians, can perform the initial triage. Additionally, all hospital staff should undergo training on how to conduct fast and effective triage. Adaptations made in real time markedly improved the ability to keep up with the triage needs caused by an unexpectedly large influx of patients on the night of August 4.

Identification of patients: Based on our experience with triage difficulties on that night, our new revised EPP now includes the use of colored jackets with labels on the back to identify each team member, including doctors, nurses, triage staff, transporters, security, media, clinical leads, resource leads, and safety leads. On the night of the disaster, the influx of volunteers, including doctors and nurses, created additional confusion as they were unsure where to report. To address this, we have appointed a nurse to act as a resource director, available 24/7 to oversee staff and supplies in accordance with the MCI. This leader has a designated location within the hospital where volunteers can report to be assigned appropriate roles.

Regarding patient identification, in our revised plan, patients will receive numbered wristbands during the initial triage process to help identify them during care and admission. Assigned staff will later collect the data during a less chaotic time to ensure proper patient identification. All patients should be identified with a unique number (using a pre-printed numbering label available in the ED EPP kit). The triage team should place a triage color (red, yellow, green, or black) on the patient’s neck or chest.

If the patient's name can be determined, the triage team will manually write the name on the wristband and the pre-printed numbered label. If the patient's name cannot be determined, the triage team will manually write the word “Unknown” on a pre-printed numbered label.

Hospital network system: The Beirut blast underscored the importance of creating an interconnected hospital network to effectively manage MCIs. In such crises, where local hospitals may be overwhelmed or incapacitated, a hospital network system would allow for seamless patient transfers between facilities, optimizing the distribution of medical care and resources.

A hospital network would consist of a centralized communication hub that connects hospitals across the region. During an MCI, this hub would monitor the status of hospitals, including available beds, ICU capacity, and the ability to perform surgeries. Hospitals in the disaster zone, once they reach capacity, could notify the hub, which would then coordinate with less affected or remote hospitals to transfer patients. This system would prevent the overburdening of certain hospitals and ensure that critical cases are transferred to facilities equipped to handle them.

Communication: Colored jackets that will be worn to identify an individual's role were introduced to facilitate communication in a chaotic environment during a disaster. In terms of safety, the challenge is to control the flow of patients, transporters, and loved ones to the emergency room. It was decided to implement this as a first step after establishing an EPP to improve security controls. The plan includes locking all hospital doors to prevent unauthorized access and maintain security during the crisis.

COVID-19 outbreak: The COVID-19 pandemic had a major impact on patient care. It compelled hospitals to establish infection control measures, such as isolation and quarantine protocols, which limited their capacity to treat the influx of injured patients caused by the explosion. Additionally, it resulted in a lack of medical supplies and manpower, making it difficult for hospitals to provide proper patient care, especially during the explosion. These challenges emphasize the necessity of disaster planning and the need for hospitals to have comprehensive emergency plans that consider the potential impact of pandemics on patient care during a disaster.

Unpreparedness and risk assessment: Faced with more complex and stressful situations than expected, healthcare professionals often feel unprepared and unsure of what to expect and what to do. No one anticipated experiencing the largest reported non-nuclear blast [7] and working under such disturbing circumstances, where half of the emergency department was filled with shattered glass and broken doors, a nonfunctional elevator, and a power outage. Since Lebanon is a country with a high risk of terror attacks, our new EPP includes a backup site for when the emergency department is attacked and is fully or partially nonfunctional.

Moreover, the importance of drills cannot be overstated. To evaluate the process and develop collaborative dynamics, it is critical to perform periodic drills with a limited group of physicians and nurses. The hospital's experience with the Beirut Port explosion demonstrated the complexities of dealing with such an MCI. As a result, it is advised to conduct two to three drills a year that exert high stress on the system, with at least one or two surprise drills for an overwhelming MCI.

Requesting tests: During MCIs, resources such as medical staff, equipment, and time are stretched thin. It is crucial to prioritize patient care efficiently to maximize the number of lives saved. One key lesson from past MCIs, such as the Beirut explosion, is that only radiological tests that directly influence patient disposition should be performed. In an MCI, radiological imaging such as X-rays, CT scans, and ultrasounds can help diagnose trauma, internal injuries, and fractures. However, performing these tests on every patient is not feasible, especially in situations where resources and time are limited.

Evacuation plan: An evacuation plan is currently under development to complement the existing disaster plan. This plan is essential for ensuring preparedness in emergency situations and is required for all hospitals. Once finalized, it will provide clear guidelines for the safe and efficient evacuation of patients, staff, and visitors during critical incidents. The evacuation plan will integrate with existing protocols to ensure comprehensive disaster response and continuity of care. The hospitals that were damaged and required quick patient evacuation during the devastating incident demonstrated the significance of having an evacuation plan.

Interdepartmental coordination: The August 4th Beirut explosion highlighted the necessity for a robust, comprehensive, and well-coordinated hospital response during an MCI. The immediate and overwhelming impact of the blast required swift action from all hospital departments. In response, the hospital's emergency plan was revised and communicated to all department heads, including radiology, laboratory, ICU, OR, and pharmacy, so that each unit could refine and adapt its own strategies in alignment with the updated ED plan.

Conclusion

The catastrophic Beirut explosion on August 4, 2020, demonstrated that even the most meticulously planned EPP can falter when faced with an unprecedented disaster of such magnitude. LAUMC’s experience sheds light on the limitations and the need for continuous adaptation of hospital disaster response systems. Key deficiencies, such as the overwhelming patient influx, breakdown in communication, and identification issues, were exposed despite the existence of an EPP. This underscores the crucial importance of conducting regular drills under high-stress conditions, assessing risks in real time, and revising preparedness plans in line with actual challenges faced during MCIs. Furthermore, the difficulties experienced with implementing the triage and patient identification systems, and effective hospital network coordination highlighted the necessity of incorporating flexible and scalable solutions into disaster plans. The post-blast revisions to LAUMC's EPP, such as the relocation of triage stations outside the ED, implementation of wristbands for patient identification, and the development of a hospital network system, offer practical insights for other institutions preparing for MCIs. This experience also emphasized the critical role of interdepartmental coordination and communication, especially when hospital infrastructure is compromised. Identifying team members via color-coded jackets, having a backup disaster site, and developing an evacuation plan are essential strategies for enhancing preparedness. The global COVID-19 pandemic further complicated the response, making it evident that pandemic scenarios must be fully integrated into disaster planning to ensure continuity of care during complex emergencies.

The key take-home message of this article is that no hospital is immune to the overwhelming challenges posed by an MCI, and existing disaster preparedness plans must be rigorously tested, revised, and expanded to reflect real-world complexities. Continual reassessment, regular drills, and a flexible response plan tailored to local vulnerabilities are paramount for improving resilience and ensuring that healthcare systems can deliver adequate care during future crises.
